# Supplementary material for: Longan Flower Ethanol Extract, Dimocarpus longan Lour, Mitigates Oxidative Damage and Inflammatory Responses While Promoting Sleep-Related Enzymes in Cell Models
Source: Biomedicines. 2025 Jun 29;13(7):1588. doi: 10.3390/biomedicines13071588 (PMC12292598; doi:10.3390/biomedicines13071588)
Supplement: Supplementary file 1 [file biomedicines-13-01588-s001.zip › biomedicines-3693906-supplementary.pdf]

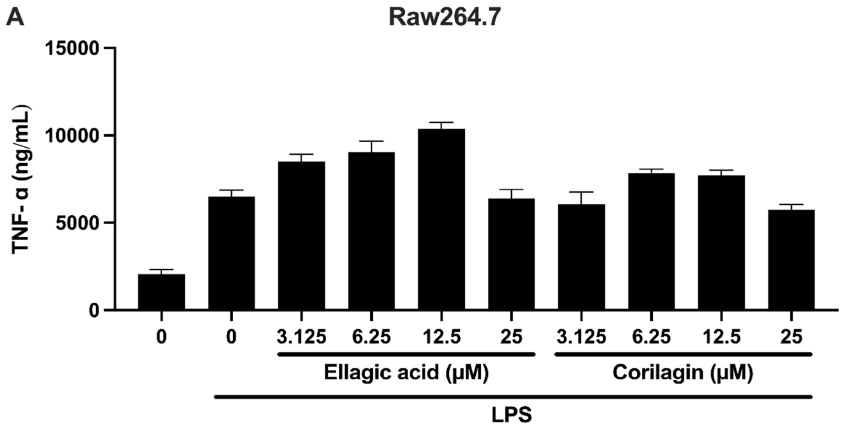

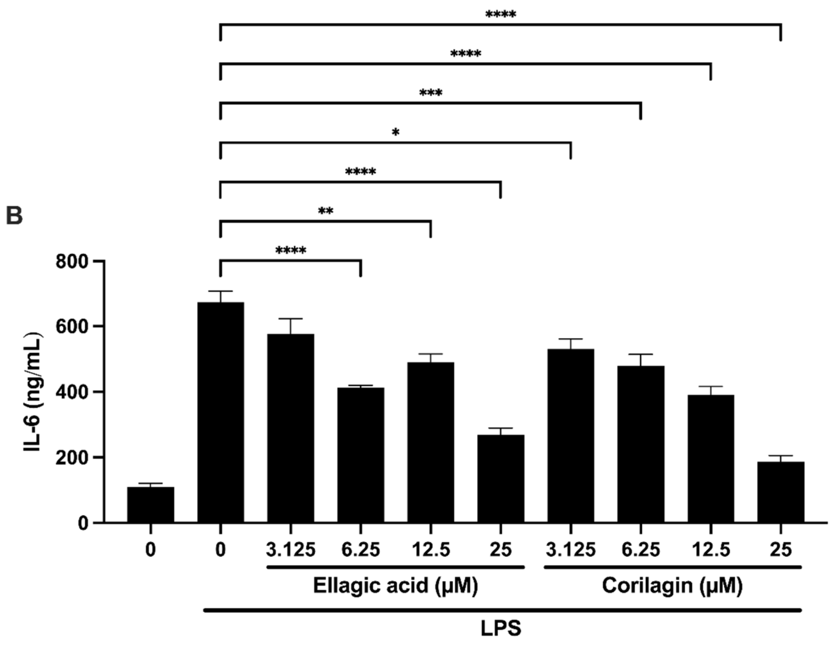

Figure S1. Suppression of inflammation by Ellagic and Corilagin in Raw264.7 cells.

The production of Ellagic and Corilagin on pro-inflammatory cytokines production were evaluated in inflammatory cell models. (A) TNF-α and (B) IL-6 production in RAW264.7 macrophages treated with LPS and varying concentrations of Ellagic or Corilagin (0-25 μM). Data are presented as mean values ± SEM (n=3). *$p < 0.05$, **$p < 0.01$, ***$p < 0.001$, ****$p < 0.0001$ (one way ANOVA with Dunnett's multiple comparisons test). LPS, Lipopolysaccharide

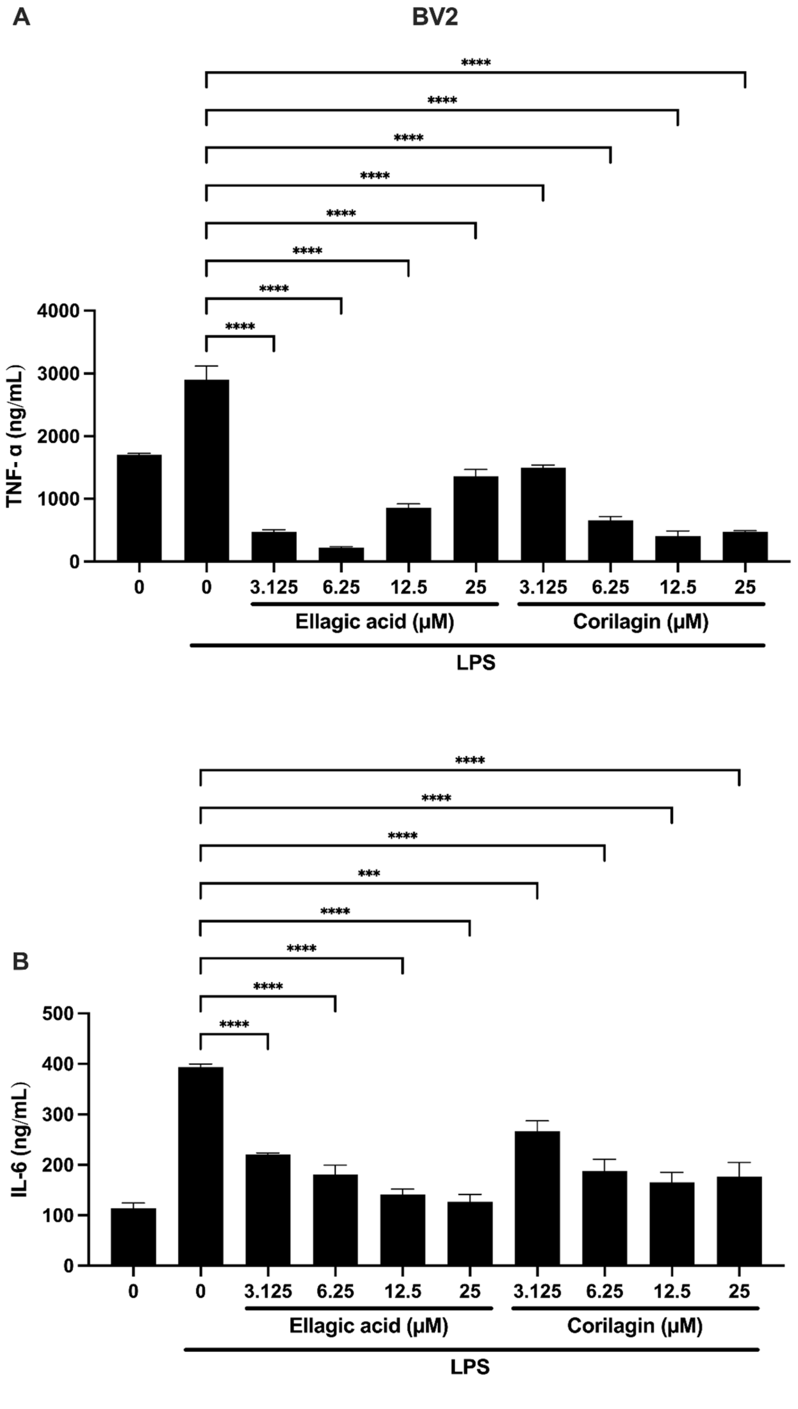

Figure S2. Suppression of inflammation by Ellagic and Corilagin in BV2 cells.

The production of Ellagic and Corilagin on pro-inflammatory cytokines production were evaluated in inflammatory cell models. (A) TNF-α and (B) IL-6 production in BV2 microglial cells treated with LPS and varying concentrations of Ellagic or Corilagin (0-25 μM). Data are presented as mean values ± SEM (n=3). ****$p < 0.001$*, ****p < 0.0001 (one way ANOVA with Dunnett's multiple comparisons test).

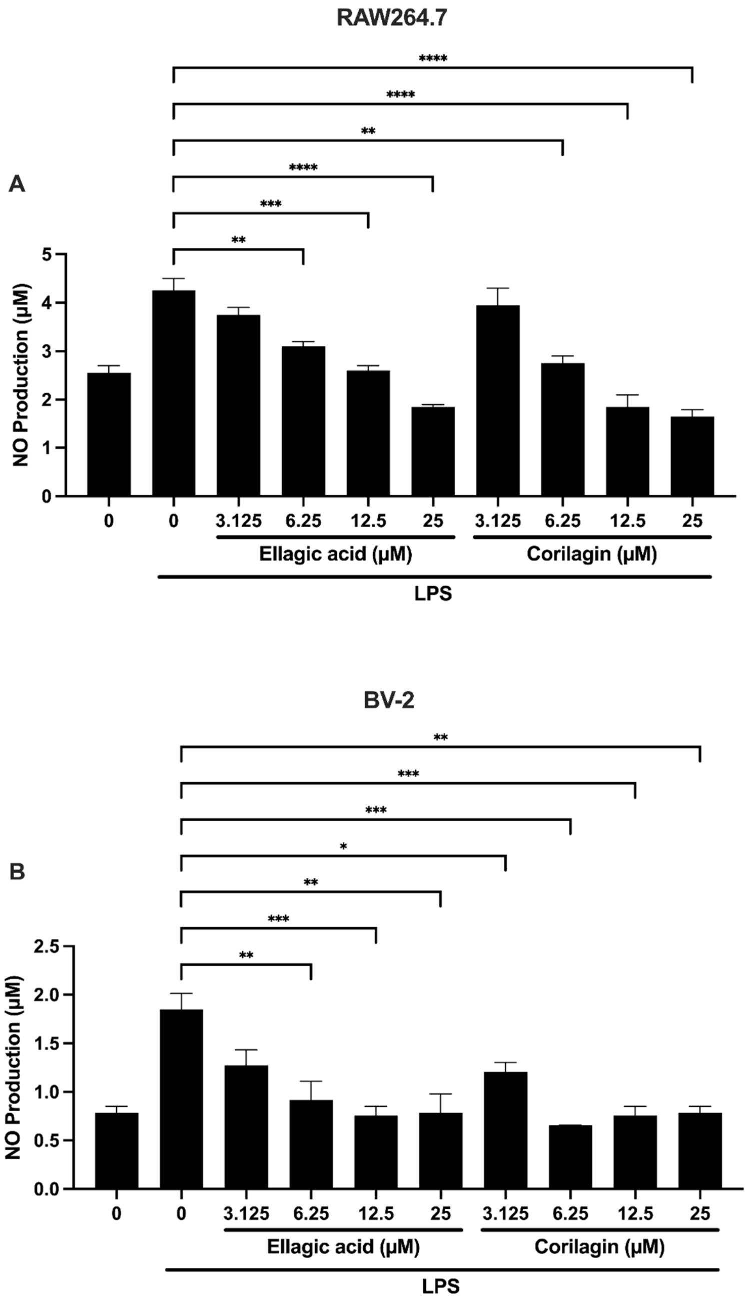

Figure 3. Suppression of Nitric Oxide Production by Ellagic and Corilagin in Immune Cells.

The effect of Ellagic and Corilagin on nitric oxide (NO) production was evaluated in inflammatory cell models. NO production in (A) RAW264.7 macrophages and (B) BV-2 microglial cells treated with LPS and varying concentrations of Ellagic or Corilagin (0-25 μg/mL). Data are presented as mean values ± SEM (n=2). *$p < 0.05$, **$p < 0.01$, ***$p < 0.001$, ****p < 0.0001 (one way ANOVA with Dunnett's multiple comparisons test).

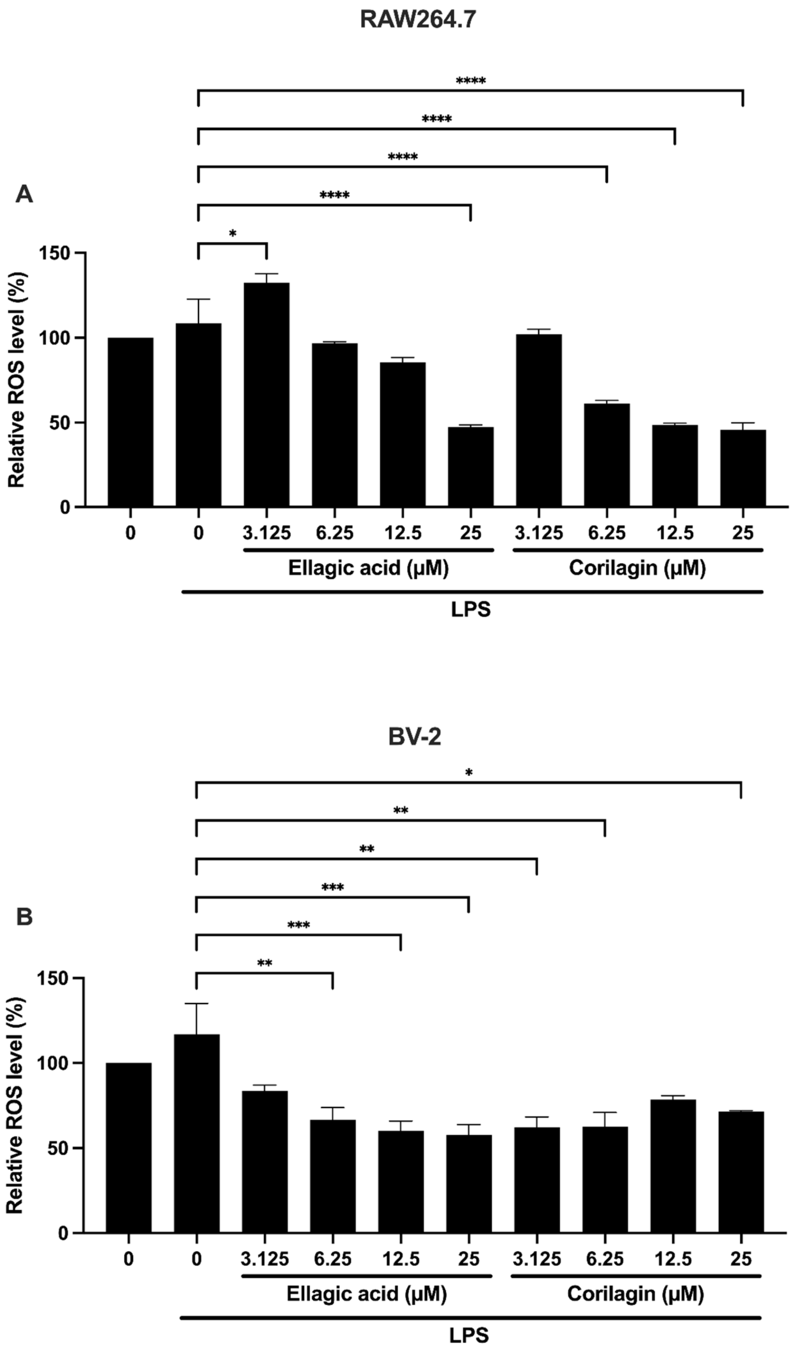

Figure 4. Suppression of ROS Production by Ellagic and Corilagin in Immune Cells.

The effect of Ellagic and Corilagin on ROS production was evaluated in inflammatory cell models. ROS production in (A) RAW264.7 macrophages and (B) BV-2 microglial cells treated with LPS and varying concentrations of Ellagic or Corilagin (0-25 μg/mL). Data are presented as mean values ± SEM (n=3). *$p < 0.05$, **$p < 0.01$, ***$p < 0.001$, ****$p < 0.0001$ (one way ANOVA with Dunnett's multiple comparisons test).
